# Effect of Blood Component Coatings of Enosseal Implants on Proliferation and Synthetic Activity of Human Osteoblasts and Cytokine Production of Peripheral Blood Mononuclear Cells

**DOI:** 10.1155/2016/8769347

**Published:** 2016-08-29

**Authors:** Lucie Himmlova, Dana Kubies, Hana Hulejova, Jirina Bartova, Tomas Riedel, Jana Stikarova, Jiri Suttnar, Vlasta Pesakova

**Affiliations:** ^1^School of Dental Medicine, General University Hospital in Prague, First Faculty of Medicine, Charles University in Prague, Karlovo Namesti 32, 121 11 Prague, Czech Republic; ^2^Institute of Macromolecular Chemistry, Academy of Sciences of the Czech Republic, Heyrovskeho Namesti 2, 162 06 Prague, Czech Republic; ^3^Rheumatological Institute, Na Slupi 4, 128 50 Prague, Czech Republic; ^4^The Institute of Hematology and Blood Transfusion, U Nemocnice 2094/1, 128 20 Prague, Czech Republic

## Abstract

The study monitored* in vitro* early response of connective tissue cells and immunocompetent cells to enosseal implant materials coated by different blood components (serum, activated plasma, and plasma/platelets) to evaluate human osteoblast proliferation and synthetic activity and inflammatory response presented as a cytokine profile of peripheral blood mononuclear cells (PBMCs) under conditions imitating the situation upon implantation. The cells were cultivated on coated Ti-plasma-sprayed (Ti-PS), Ti-etched (Ti-Etch), Ti-hydroxyapatite (Ti-HA), and ZrO_2_ surfaces. The plasma/platelets coating supported osteoblast proliferation only on osteoconductive Ti-HA and Ti-Etch whereas activated plasma enhanced proliferation on all surfaces. Differentiation (BAP) and IL-8 production remained unchanged or decreased irrespective of the coating and surface; only the serum and plasma/platelets-coated ZrO_2_ exhibited higher BAP and IL-8 expression. RANKL production increased on serum and activated plasma coatings. PBMCs produced especially cytokines playing role in inflammatory phase of wound healing, that is, IL-6, GRO-*α*, GRO, ENA-78, IL-8, GM-CSF, EGF, and MCP-1. Cytokine profiles were comparable for all tested surfaces; only ENA-78, IL-8, GM-CSF, and MCP-1 expression depended on materials and coatings. The activated plasma coating led to uniformed surfaces and represented a favorable treatment especially for bioinert Ti-PS and ZrO_2_ whereas all coatings had no distinctive effect on bioactive Ti-HA and Ti-Etch.

## 1. Introduction

Bone/implant healing process can be enhanced and accelerated by a mechanical/physicochemical [1–3] and/or biological treatment of the surface [4]. The biological treatment includes the surface treatment with proteins of extracellular matrix such as fibrinogen/fibrin, collagen and other fibrous proteins, glycoproteins (e.g., fibronectin, laminin), glycosaminoglycans, growth factors (e.g., bone morphogenetic proteins), or specific peptide sequences (e.g., RGD-based sequences). The detailed overview about the particular approaches can be found in several reviews [5–8]. The biological modification also includes a treatment with the complex media such as platelet-rich plasma (PRP) [9–14] or whole blood [9].

The blood component treatment is based on the fact that blood is a site of the first contact between the foreign implant material and the living tissue of a recipient [15, 16] and that blood supply is crucial for coagulation and wound healing [15]. Platelets perform many functions, including formation of a blood clot and release of growth factors (GF) into the wound. The GFs (e.g., platelet derived GFs, transforming GF beta, BMPs, or insulin-like GF) assist the body in repairing itself by stimulating stem cells to regenerate a new tissue. The platelet-rich plasma (PRP) defined as a high concentration of autologous platelets in a small volume of autologous plasma [17] represents a natural source of growth factors [18], but its effect on the bone/implant healing process is still controversial [7, 12, 14, 18–22]. The reasons for these discrepancies may consist in differences in the activation protocol [12] or study design [10, 11]. An additional reason could be also the immune reaction between human and animal proteins where an animal source of additive components (e.g., bovine thrombin as a PRP activator or anticoagulants) is used [17, 23]. Bovine thrombin has been used mainly in earlier studies and procedures as a “gold standard” for platelet activation in PRP [17] and currently is often replaced by autologous thrombin. Nevertheless, alternative ways to activate PRP by thrombin receptor activating peptide (TRAP) or pulse electric field (PEF) to prevent the abovementioned immune response are under investigations [23]. Albumin is the main human blood plasma protein [24] with many functions. Among others, albumin serves as a carrier for molecules (hormones, salts, drugs, etc.) [25, 26]. Albumin is also a well-known proliferative factor in the cell culture that can improve the remodeling characteristics of grafts [27, 28]. Fibrin is the last step in the coagulation cascade and plays an overlapping role in blood clotting, fibrinolysis, cellular and matrix interactions, inflammatory responses, and wound healing [15, 29, 30]. Fibrin has long been successfully used as a fibrin glue/sealant [30, 31]; nowadays, fibrin is mostly used for fibrin scaffolds [32, 33]. Fibrin also serves as a carrier in the platelet poor plasma (PPP) and PRP treatments after their activation [20] and in a platelet-rich fibrin (PRF) protocol, which are currently used during surgical procedures [9, 10, 12, 20, 34]. Fibrin's biological activity cannot be neglected because of its role in the activation of innate immunity during wound healing [35].

The place of the first contact between an implanted foreign material and living tissue of a recipient is blood. The wide scale of treatment options mentioned above, discrepancies in study results [7, 9, 11, 12, 14, 19, 21–23], and a change in the approach to the osseointegration definition in the light of present findings [36] made us evaluate the cell behavior in the immediate vicinity of the implant in presence of various blood components* in vitro* as an important step towards better understanding of the healing processes. In the present study, the basic cellular* in vitro* model for the cell/implant integration study was extended by an introduction of a biological layer of blood proteins (albumin, fibrin network) and/or platelets on the implant surface. This experimental arrangement would better imitate the situation upon implantation at the initial phase of the healing process [15] and enables evaluating the effect of the particular blood components on osteoblast cells and on cytokine production of peripheral blood mononuclear cells (PBMCs) in* in vitro* conditions. Normal human osteoblasts were cultivated on four commercially available implant materials without/with blood component coatings (i.e., serum, activated plasma, and plasma/platelets). The proliferation and synthetic activity of the cells were evaluated with respect to the physicochemical surface properties or the biological coating. The adhesion and activation of platelets on the pristine implant surfaces were monitored as well. Peripheral blood mononuclear cells were used for monitoring the immune response to the biological coating type because implant healing is an immunological and inflammation-driven process [36].

## 2. Materials and Methods

### 2.1. Materials and Sterilisation

The following commercially available materials were selected for the study: titanium with an etching surface treatment (Ti-Etch, Fopos, Praha, Czech Republic), plasma-sprayed titanium (Ti-PS, Beznoska, Kladno, Czech Republic), titanium with plasma-sprayed hydroxyapatite (Ti-HA, Lasak, Prague, Czech Republic), and ZrO_2_ ceramics (ZrO_2_, Saint Gobain, Turnov, Czech Republic). All the surface treatments (etching, Ti/hydroxyapatite plasma-spraying) were provided by the manufacturer. Tissue-culture-grade polystyrene (TCPS, Nunc A/S, Roskilde, Denmark) represents a material with properties optimised for the cultivation of eukaryotic cells* in vitro* and was used as a positive control.

For osteoblast cultivation, round-shaped substrates of 20 mm in diameter with a thickness of 1 mm were used. Square plates (20 × 10 × 1 mm) were used to measure the surface free energy, wettability, and surface roughness values. Samples were washed in deionised water and ethanol using an ultrasonic bath and subsequently autoclaved before characterization of the surface properties and cell cultivations.

### 2.2. Biological Coatings of Implant Materials

The round-shaped samples were biologically coated with serum (a model for albumin), the activated plasma (a model for fibrin network), or a platelet-rich plasma (plasma/platelets, a model for platelets) under sterile conditions prior to cell inoculation.

#### 2.2.1. Coating with Serum

The samples were incubated with human serum (Cambrex, Bio Science, USA) for 60 minutes at the laboratory temperature. Then, the excess serum was drawn off and replaced with a sterile saline solution (0.9% wt solution of NaCl, pH = 7.4).

#### 2.2.2. Coating with Activated Plasma

A frozen human citrate-phosphate-dextrose stabilized blood plasma (CPD, IHBT, Prague, Czech Republic) was defrosted in a water bath at 37°C. Then, calcium chloride (1.6 g of CaCl_2_ per liter of CPD) was added to CPD to debond the stabilization citrate and activate the coagulation cascade (e.g., formation of a fibrin network). The samples were immersed in the activated plasma for 6 minutes (see below for the clotting time). Then, the plasma was gradually drawn off and a sterile saline solution was added simultaneously to prevent blood clot formation. After plasma replacement, the samples were rinsed 3 times with a sterile saline solution and placed into cultivation wells.

The clotting time (6 minutes) was estimated by a turbidity measurement on the TCPS control surface (Figure S1, supplementary information in Supplementary Material available online at http://dx.doi.org/10.1155/2016/8769347) according to the following protocol: calcium chloride (10 mM) was added to the freshly thawed CDP plasma. 1 mL of the solution was immediately transferred to the polystyrene cuvette (1 cm path length) for measurements. The intensity changes of the light passing through the cuvette were measured at 350 nm in two-minute intervals at the room temperature using a spectrophotometer (Biochrom Libra S22, Cambridge, UK).

#### 2.2.3. Coating with Plasma/Platelets

The fresh platelet-rich plasma (PRP) was prepared immediately before each experiment from the blood of a healthy donor as follows: the blood was taken under sterile conditions, stabilized with sodium citrate (1 mL to 9 mL of blood), and centrifuged according to a standard protocol. The platelet concentration in the PRP (Coulter Counter Onyx, Coultronix) was adjusted to a value of 250 000 platelets/*μ*L by dilution with a pure plasma from the same donor. The samples were incubated with 1 mL of PRP for 1 hour at the laboratory temperature. Then, the PRP was drawn off and the samples were washed 3 times with a sterile saline solution, pH = 7.4. This group was named as a plasma/platelets group.

### 2.3. Surface Characterization

*Surface free energy γ* was estimated by measuring static contact angles (*θ*) in three different solvents (water, formamide, and diiodomethane) using the sessile drop method (video-camera-based OCA 20 instrument, Dataphysics, Filderstadt, Germany). The average value of *θ* obtained from the Young-Laplace fitting approach was used to calculate the surface free energy (*γ*) and its polar (*γ* ^*P*^) and disperse (*γ* ^*D*^) components using the OWCK method [37].

*Surface wettability* was determined by measuring advancing (*θ*_*A*_) and receding (*θ*_*R*_) contact angles by the dynamic Wilhelmy plate method (tensiometer Kruss K12, Hamburg, Germany) in water.

*Surface roughness*, represented here by the mean arithmetic average of the profile height *R*_*a*_, was measured using the Hommeltester 1000 T device (Hommelwerke, Germany).

*Surface topography* was observed using a scanning electron microscope (VEGA Plus TS 5135, Tescan s.r.o., Brno, Czech Republic). The samples with the biological coating with activated blood plasma or platelet-rich plasma (see Section 2.2) were prepared in parallel with the cell cultivation samples. After coating, samples were transferred through ethanol series to pure ethanol and dried.

### 2.4. Adhesion of Platelets to Implant Materials and Determination of Serotonin

The platelet-rich plasma (PRP) for the platelet adhesion analysis and the serotonin analysis was prepared in the same way as the PRP for osteoblast activity monitoring (see Section 2.2). The number of platelets in PRP was adjusted to 250,000/*μ*L.

First, six-well cultivation dishes (Nunc A/S, Roskilde, Denmark) were saturated with a 1% wt solution of bovine serum albumin (BSA) in the PBS buffer, pH = 7.4, for 1 hour and then rinsed 3 times with PBS. Then, the pristine round-shaped Ti-HA, Ti-Etch, Ti-PS, and ZrO_2_ samples (three parallel samples for each surface) were placed into the saturated wells and 1 mL of PRP was carefully applied on the sample surface. After one-hour incubation under static conditions at the laboratory temperature, the rest of PRP was gently aspirated, 250 *μ*L of the mixture was centrifuged at 3000 ×g for 2 min at 20°C, and 100 *μ*L of supernatant was prepared for the serotonin analysis. The analysis of serotonin (5-hydroxytryptamine, 5-HT) released by platelets was performed chromatographically according to Křížová et al. [38].

The plasma/platelets-coated samples were then transferred to clean dishes and rinsed 10 times with PBS. The amount of the adhered platelets was calculated via determination of the acid phosphatase activity [39, 40]. Briefly, the samples were placed with the coated side downwards into clean dishes filled with 235 *μ*L of 5 mM* para*-nitrophenylphosphate and 1% Triton X-100 in 0.1 M citrate buffer, pH 5.4, and left to react for 1 hour. The reaction was terminated by addition of 165 *μ*L of 2 M NaOH and shaking for 2 min. Then, 175 *μ*L of the final solution was added directly into the 96-well plate and absorbance was measured with a microplate reader CERES 900 (Biotek Instruments, USA) at 405 nm against a platelet-free blank. The amount of adhered platelets was calculated using a calibration curve estimated in standard suspensions of platelets. A linear relationship exists between the optical density at 405 nm and the cell number in the used calibration range. The data were expressed as a percentage of adhesion against the positive TCPS control.

### 2.5. Osteoblast Cultivation and Proliferation

Human osteoblasts (NHOst, Cambrex Bio Science Walkersville, Inc., USA) were cultivated up to the 3rd passage. The samples were put into a six-well dish (Nunc A/S, Roskilde, Denmark). Osteoblasts were inoculated on the surfaces at a density of 15,000/cm^2^ and cultivated in the OGM BulletKit medium (Cambrex Bio Science Walkersville, Inc., USA) at 37°C and 5% CO_2_ for 6 days. Osteoblast differentiation was monitored using the OGM Differentiation SingleQuots set (Cambrex Bio Science Walkersville, Inc., USA). The harvested cells and culture medium were used for further analysis.

Osteoblast proliferation was determined from the mitochondrial oxidising activity of the cellular monolayer cultivated on the tested materials using the Laughton MTT test [41] six days after the cultivation. In parallel, the same MTT test was performed for the concentration series of osteoblasts adhered to TCPS; in this way, a calibration curve was obtained [42]. Osteoblast cultivation was repeated in three independent experiments. Every time, three parallel samples were tested for a particular surface. Cell cultivation on TCPS with the corresponding biological surface treatment (i.e., pristine, serum/activated plasma/plasma/platelets-coated TCPS surfaces) was used as a control for all determinations.

The cell number determined in the TCPS controls was set up as a 100% reference value in each independent experiment. Then, data for each surface were expressed in % of the corresponding TCPS control. This approach allowed for a comparison of results between three independent experiments and simultaneously between different biological coatings.

### 2.6. Synthetic Activity of Osteoblasts

The osteoblast synthetic activity was monitored by means of mediators expressed by cells to the cultivation medium after 6-day cultivation. The following cytokines were selected: (a) bone specific alkaline phosphatase (BAP) as a biochemical marker of bone metabolism and osteoblast activity [43]; (b) receptor-activator of the nuclear factor- (NF-) kB ligand (RANKL) as a marker of osteoclast differentiation [44]; (c) interleukin-8 (IL-8) as an indicator of inflammatory reaction, that is, inflammatory chemokine increasing the expression of adhesive molecules on the cell surface [45]. Cytokine expression was determined for each surface in three independent experiments with three parallel samples. These three parallel samples were pooled and subsequently processed as one sample by the ELISA analysis (Bender Company, MedSystems, Vienna, Austria).

The data obtained from ELISA were recalculated to be relative for the cell number determined on the particular surface (see Section 2.5). The following units were used: U/L/100,000 cells for BAP, ng/mL/100,000 cells for IL-8, and pmol/L/1,000,000 cells for RANKL. The cytokine expression in the TCPS controls with the corresponding biological surface treatment (i.e., pristine and serum, activated plasma, and plasma/platelets-coated TCPS surfaces) was used as a control.

The cytokine expression determined in the TCPS controls was set up as a 100% reference value for each cell cultivation. Then, data for each surface were expressed in % of the corresponding control TCPS surface. This approach allowed for a comparison of results between three independent experiments and simultaneously between different biological coatings.

### 2.7. Cultivation and Synthetic Activity of Human Peripheral Blood Mononuclear Cells

Human peripheral blood mononuclear cells (PBMCs) were separated by gradient centrifugation (Histopaque, Sigma-Aldrich, St. Louis, USA) from a buffy coat obtained from the Institute of Hematology and Blood Transfusion (IHBT, Prague, CR). The buffy coat was diluted with an X-Vivo tissue medium (Cambrex Bio Science, Walkersville, Inc., USA) in the 1 : 1 ratio and, after centrifugation, the PBMC sediment was rinsed and diluted with an X-Vivo medium to a concentration of 10^6^ cells per mL.

Two parallel samples of each tested surface were placed in a 24-well dish. Peripheral blood mononuclear cells were inoculated onto the samples at the dose of 2 × 10^6^ cells per 2 mL and cultivation was performed at 37°C in a 5% CO_2_ atmosphere for 5 days. After cultivation, the supernatants were collected; every 2 parallel identical samples were pooled and frozen at −20°C. Cytokine production was assessed using a commercially available RayBio Human Cytokine Antibody Array 3 analysis (AAH-CYT-3, RayBiotech, Inc., Norcross, USA). The list of the selected cytokines is presented in Figure S2. The method provides semiquantitative imaging of cytokine and protein spectra using coloring density of the membrane-based antibody arrays. Detection was performed using a luminescence detector Fujifilm LAS 1000. The obtained data were digitalized and image analysis (AIDA 3.28, Ray test, Straubenhardt, Germany) was performed. The positive and negative controls given by the supplier for each membrane served as the maximum/minimum density gauges. The obtained results are presented as a percentage of the positive control, which represents 100%.

### 2.8. Statistical Analysis

Five plates of each material were used for surface free energy (*γ*) evaluation; every sample was characterized by 6 drops of the used solvent (see Section 2.3). Five parallel samples of each material were used for all dynamic contact angle measurements. Two parallel samples of each material were used for surface roughness evaluation; the measured values were processed using standard statistical tools (mean, significant deviation).

The MTT test for proliferation assay was performed for each independent cell experiment, always with three parallel samples for each surface type. Three independent experiments were performed. The results were statistically analyzed using the *t*-test (*p* < 0.05) and ANOVA test.

The expression of cytokines produced by osteoblasts (BAP, RANKL, and IL-8) was determined in each of three independent cell experiments. Three parallel samples of each surface were pooled and subsequently processed as one sample (mean, significant deviation).

The cytokine expression by human peripheral blood mononuclear cells (RayBio Human Cytokine Antibody Array 3 set) was determined for the each pristine and corresponding biologically coated surface. The analyzed samples represented pooled samples from three independent cultivations with three parallel samples for the surface. Data were expressed in % versus the positive control of the array (100%).

## 3. Results

### 3.1. Surface Characterization

The determined values of monitored surface parameters, that is,* surface free energy γ* and its polar (*γ* ^*P*^) and disperse (*γ* ^*D*^) components,* contact angles*, and* roughness*, are given in Table 1. The tested materials are arranged according to decreasing *γ* ^*P*^, which was considered to be an important factor affecting biological interactions [46, 47].

Significant differences were observed in *γ* for the studied surfaces. The calculated *γ* varied from 7.23 to 70.21 mN/m. The highest values of *γ* and *γ* ^*P*^ were measured for the TCPS control. Calculation of *γ* was not performed for Ti-PS due to its high surface roughness value. However, Ti-PS was placed after TCPS in Table 1 for the following reason: considering that *γ* is determined by surface properties based on physicochemical interactions on the atomic level, the surface free energy values obtained for the polished titanium in the previous project, *γ* = 47.01 ± 1.54 (mN/m), *γ* ^*P*^ = 15.97 ± 0.54 (mN/m), and *γ* ^*D*^ = 31.05 ± 0.98 (mN/m), could be applied approximately to Ti surfaces with variable roughness values [48]. The deposition of an upper inorganic hydroxyapatite layer on Ti (Ti-HA) resulted in a surface with extremely low *γ* (7.23 ± 3.05 mN/m) and *γ* ^*D*^ values, but with *γ* ^*P*^ equal to 4.62 mN/m.

*Surface wettability* has been considered one of the main factors reflecting the degree of cell adhesion to artificial surfaces. TCPS, Ti-Etch, and ZrO_2_ ceramics represent surfaces with moderately high wettability (*θ*_*A*_ under 70°). Ti-PS is a very hydrophobic surface (*θ*_*A*_ of 110°). The observed low wettability of the Ti-PS surface compared with the hydrophilic polished Ti surface (*θ*_*A*_ of 62°) [3] is a result of a high surface roughness. Nonmetal Ti-HA is a hydrophobic surface with *θ*_*A*_ over 90°. For detailed comments concerning the surface properties, refer to our previous work [48].

**Figure 1:**
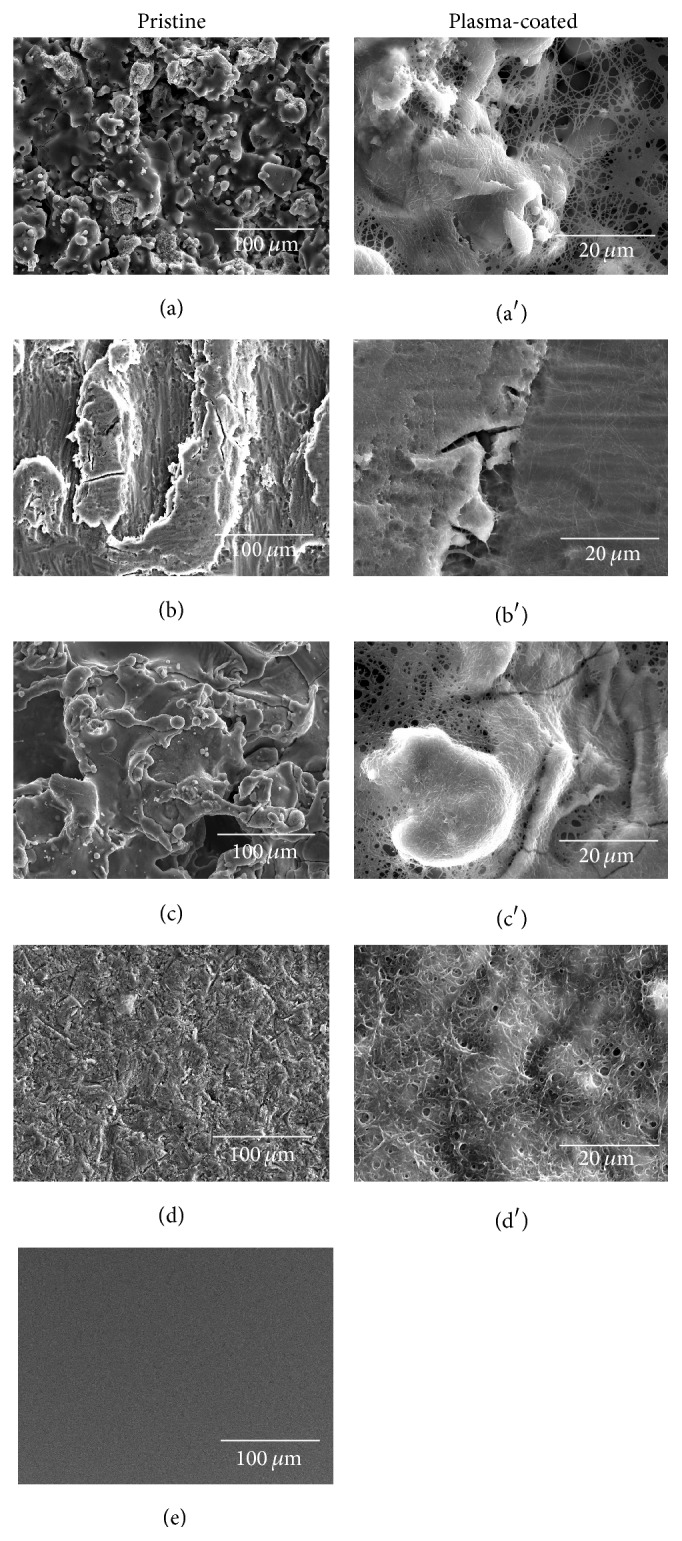
SEM analysis of the pristine implant materials Ti-HA (a), Ti-Etch (b), Ti-PS (c), ZrO_2_ ceramic (d), and control TCPS (e) and the surfaces coated with the activated plasma Ti-HA (a′), Ti- Etch (b′), Ti-PS (c′), and ZrO_2_ ceramic (d′).

The pristine surfaces and surfaces coated with the activated plasma, that is, with a fibrin network formed by fibrin cross-linking after debonding of the stabilization citrate present in the CPD plasma, are presented in Figure 1. The pictures show that smoother Ti-Etch and ZrO_2_ surfaces were coated with a highly dense layer of a fibrin network matching the surface profile whereas the fibrin network on rough Ti-HA and Ti-PS is inhomogeneous with a clearly visible fibrous structure bridging the depressions on the surface and with 5–10 *μ*m “pores.”

### 3.2. Osteoblast Proliferation

**Figure 2:**
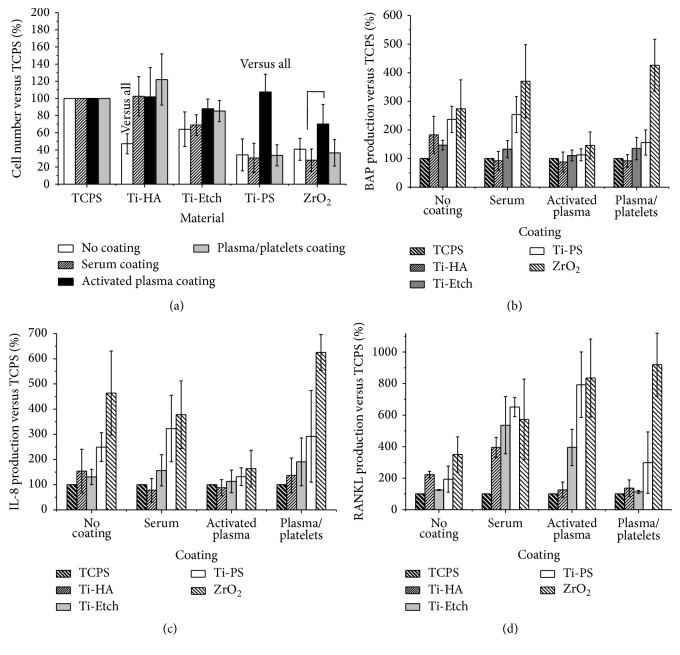
Cell proliferation (a) and production of BAP (b), IL-8 (c), and RANKL (d) by human osteoblast cells cultivated on the pristine Ti-HA, Ti-Etch, Ti-PS, and ZrO_2_ surfaces and on the surfaces coated with serum, activated plasma, and plasma/platelets. Data are expressed in %  ±SD versus the TCPS controls (100%) from three independent cultivation experiments.

Osteoblast proliferation after 6-day cultivation was monitored on the pristine surfaces and surfaces coated with a biological layer. The biological layer was formed using three model systems: serum (i.e., albumin), activated blood plasma (i.e., a fibrin network), and plasma/platelets (i.e., plasma proteins with platelets). First, proliferation was calculated as the number of cells per mL using a calibration curve [3]. Then, the cell number determined in the TCPS controls was set up as a 100% reference value and proliferation on the pristine and coated surfaces was expressed as % of the control surface (see Section 2.5). The data are summed up in Figure 2(a) from the viewpoint of the chemical origin of the pristine surface.

Cell proliferation on all the* pristine surfaces* was statistically significantly lower than on the TCPS control surfaces (*p* < 0.05), ranging from 35 to 65% of TCPS. Proliferation increased in the Ti-PS → ZrO_2_→ Ti-HA → Ti-Etch line, but no statistically significant deviation was found between the surfaces (*p* = 0.05).

Generally,* biological coatings* increased osteoblast proliferation on almost all the surfaces, but to a different extent. All the three biological coating types supported cell proliferation on the osteoconductive Ti-HA and Ti-Etch surfaces. Proliferation on the plasma-coated Ti-HA even reached the values of the TCPS control and this increase was statistically significant for the pristine Ti-HA as well. Osteoblasts proliferated to the highest extent also on the activated plasma-coated Ti-Etch surfaces, but with no significant differences compared to the serum and plasma/platelets coatings or the TCPS control. In the case of the biotolerant Ti-PS and ZrO_2_, the serum and plasma/platelets coating yielded a proliferation rate comparable to the pristine surfaces and only the activated plasma coating induced a significantly higher osteoblast proliferation than that observed on the pristine materials.

### 3.3. Synthetic Osteoblast Activity

The expression of BAP (early osteoblast differentiation marker), RANKL (stimulation factor for osteoclast maturation, indicating bone resorption and remodeling), and IL-8 (an inflammatory chemokine) was determined in a cultivation medium after 6-day cultivation on the pristine and biologically coated surfaces using ELISA. To compare data from three independent experiments, the production evaluated for the TCPS controls was set up as a 100% reference value and cytokine production on the tested surfaces was expressed in % of the controls. The data are presented in Figures 2(b), 2(c), and 2(d) from the viewpoint of the biological coating.

The expression of investigated markers secreted by osteoblasts cultivated on all the* pristine surfaces* was higher than that on TCPS and had an increasing tendency in the Ti-Etch → Ti-HA → Ti-PS → ZrO_2_ order. Overall, the indicative average expression of cytokines reached approximately 150% on Ti-Etch, 180% on Ti-HAP, 240% on Ti-PS, and 350% on ZrO_2_ with respect to the TCPS reference.

A similar trend was observed in increased cytokine production in the order as that on the pristine surfaces for all the three* biological coatings*. All the coatings lowered* BAP production* on Ti-HAP to approximately 90% in comparison with 180% observed for the pristine surface. The BAP production on all the coated Ti-Etch surfaces was comparable to pristine Ti-Etch (130–140%). In the case of the bioinert Ti-PS, the plasma/platelets and especially the activated plasma coating reduced BAP production from 240% on the pristine surface to close above 100%. Finally, the serum and plasma/platelets coatings on ZrO_2_ resulted in a higher BAP production reaching even 400%, whereas the activated plasma coating decreased BAP production to 150% when compared with 270% on pristine ZrO_2_.

All the coatings lowered* IL-8 production* on Ti-HA to values close to 100% when compared to the pristine surface (150%). IL-8 production remained almost unchanged on the all Ti-Etch surfaces (120–150%); a noticeable increase to 190% was detected on the plasma/platelets-coated Ti-Etch. The serum coating on Ti-PS induced a one-third higher IL-8 expression than the pristine surface (250%) while the activated plasma reduced it to 130%. Finally, the plasma/platelets coating elevated IL-8 production on ZrO_2_ to 600% in comparison to 460% determined for pristine ZrO_2_ and the activated plasma decreased it to 160%.

*RANKL production* was markedly increased on all the serum and/or activated plasma-coated samples (with the exception of Ti-HA). On the other hand, the plasma/platelets coating led to a similar RANKL expression when compared to the pristine surfaces. The exception is ZrO_2_ ceramics with the highest RANKL expression detected.

### 3.4. Platelet Adhesion

**Figure 3:**
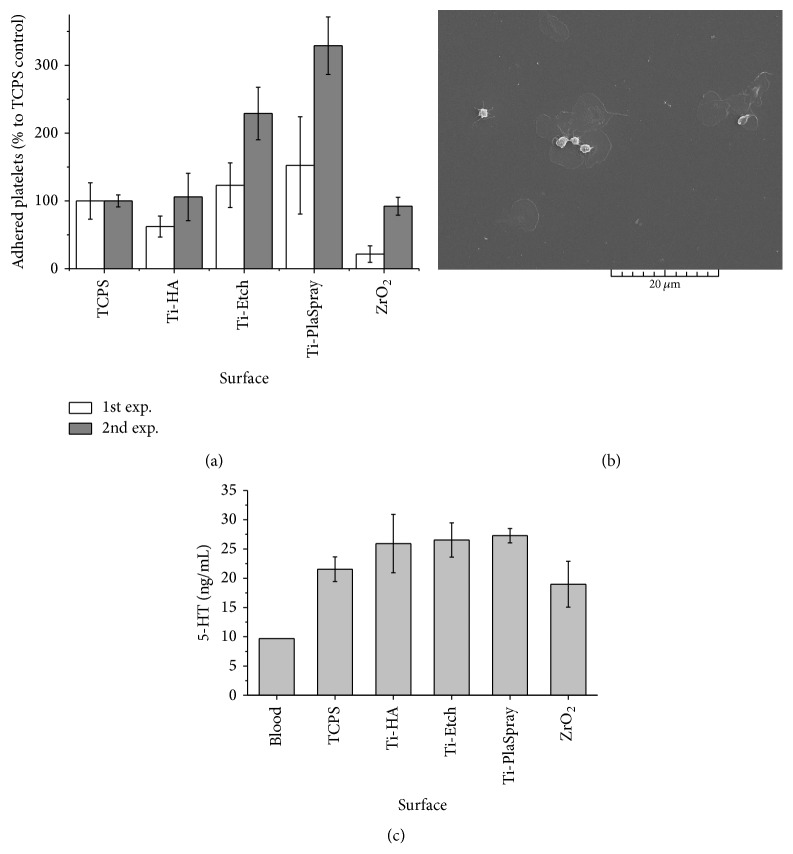
Adhesion of platelets on the pristine Ti-HA, Ti-Etch, Ti-plasma-sprayed, and ZrO_2_ surfaces (a) (acid phosphatase activity determination; two independent experiments, the platelet number on the TCPS control was 4 900 platelets/cm^2^ for experiment 1 and 470 platelets/cm^2^ for experiment 2). SEM photograph of TCPS with the plasma/platelets coating (b). The outlined shapes correspond to adhered and spread platelets; the round-shaped white objects represent aggregated platelets. The platelet activation expressed as a serotonin production (c) (HPLC analysis, 5 parallel samples).

The platelet adhesion from the PRP was evaluated on the pristine surfaces by determination of acidic phosphates produced by the platelets.  Figure 3(a) depicts the results of two representative experiments. Since the platelet activity is different for different blood donors, the number of adhering platelets was expressed in percentage against the TCPS control (set up as 100%). This recalculation allowed for an indicative comparison between the experiments.

The number of adhered platelets increased in the ZrO_2_, Ti-HA, Ti-Etch, and Ti-PS order in both the independent experiments. The platelet adhesion to Ti-HA and Ti-Etch was close to the TCPS control, while it decreased rapidly on the ZrO_2_ ceramics. The rapid decrease observed on ZrO_2_ might suggest retarded platelet aggregation after implant introduction into a tissue damaged during implantation. The most profound platelet adhesion was observed on Ti-PS with the highest surface roughness.

Figure 3(b) depicts a representative SEM photograph of the plasma/platelets-coated TCPS surface. The outlined shapes correspond to adhering platelets with various levels of activation. This means that, following adhesion to the implant surface, the round-shape platelet, if activated, changes its shape and spreads out over the surface. The round white objects observed on the photograph are aggregated platelets. Because of the surface unevenness of all the materials under the study, it was not possible to visualize the platelets in a quality suitable for publication.

All the tested surfaces activated platelets; the serotonin production was always higher than that detected in the blood (Figure 3(c)). The serotonin levels on Ti-HA, Ti-Etch, and Ti-PS were comparable and 2.5–2.8 times higher than the production in the blood. The lowest platelet activation was observed on the ZrO_2_ surface, where the serotonin production was even lower than on the control TCPS surface.

### 3.5. Synthetic Activity of Human Mononuclear Cells

**Figure 4:**
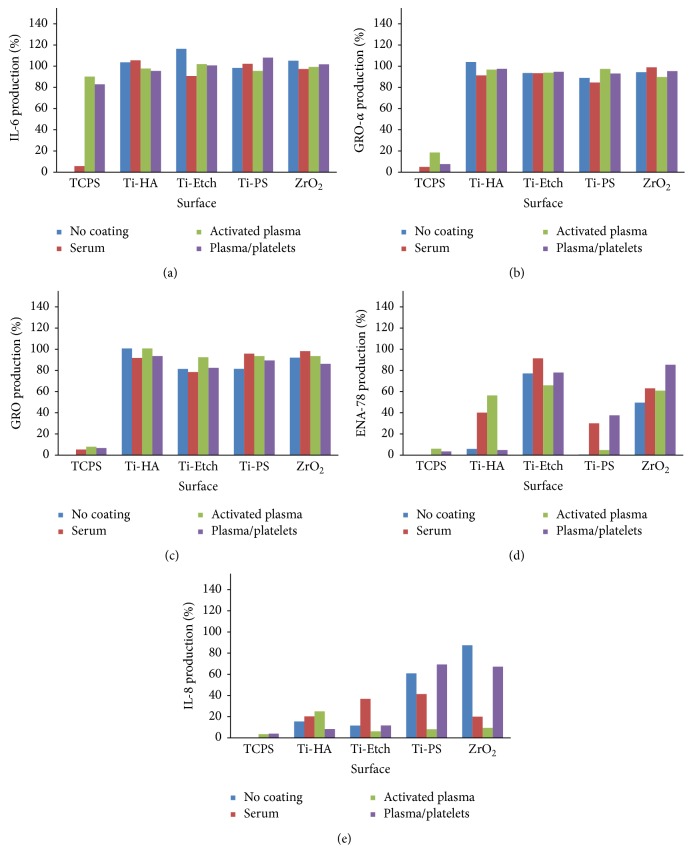
Expression of inflammatory cytokines IL-6 (a), GRO-*α* (b), GRO (c), ENA-78 (d), and IL-8 (e) by PBMCs cultivated on the pristine surfaces and the surfaces coated with serum, activated plasma, and plasma/platelets (RayBio Human Cytokine Antibody Array 3 analysis). Data are expressed in % versus the positive control of the array (100%).

**Figure 5:**
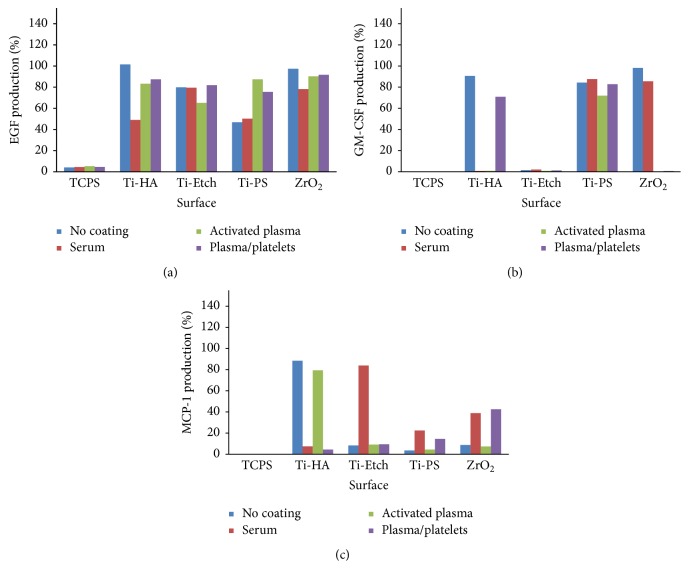
Expression of cytokines EGF (a), GM-CSF (b), and MCP-1 (c) by PBMCs cultivated on the pristine surfaces and the surfaces coated with serum, activated plasma, and plasma/platelets (RayBio Human Cytokine Antibody Array 3 analysis). Data are expressed in % versus the positive control of the array (100%).

Production of a broad spectrum of cytokines by human peripheral blood mononuclear cells (hPBMCs) cultivated on pristine and coated (serum, activated plasma, and plasma/platelets) surfaces was assessed in the cultivation medium using the RayBio Human Cytokine Antibody Array 3 analysis. Figures 4 and 5 present the obtained data expressed as a percentage of the positive control of the array (set up as 100%). The spectrum of expressed cytokines (cytokine profiles) depended on the implant material and the surface coating type. Among all the 40 selected cytokines (Figure S2), only the cytokines IL-6, GRO-*α*, GRO, ENA-78, IL-8, GM-CSF, EGF, and MCP-1 were produced on almost all the pristine, coated, and control TCPS surfaces. Further, no typical triggers of inflammation (e.g., IL-1*β*, TNF-*α*) or anti-inflammatory cytokines (e.g., IL-10) were identified. PDGF was produced only on the activated plasma-coated Ti-HA and plasma/platelets-coated Ti-PS surfaces. The other tested cytokines were produced in amounts close to the negative control and were omitted.

Generally, cytokine expression was always the lowest on the TCPS control and represented several % of the positive control of the array. The exception is a high IL-6 production (80–90%) on the TCPS surfaces coated with the activated plasma and plasma/platelets. The expression of IL-6 cytokine and GRO-*α* and GRO chemokines on all the pristine and coated surfaces reached more than 80% of the positive array control (Figures 4(a), 4(b), and 4(c)).

The ENA-78 (CXCL5) chemokine production was high on the moderately hydrophilic Ti-Etch (65–80%) and ZrO_2_ ceramic (app. 60%) surfaces, both the pristine and biologically coated (Figure 4(d)). On the contrary, the production on rough pristine hydrophobic Ti-HA and Ti-PS was very low and only slightly increased on the serum (30–40%) and activated plasma (50%) coated surfaces. The plasma/platelets coating on Ti-PS increased the ENA-78 production as well (40%).

Production of the IL-8 chemokine (CXCL8) on the pristine osteoconductive Ti-HA and Ti-Etch surfaces was only slightly increased (10–15%) to those low values observed on TCPS (Figure 4(e)); no biological coating (serum, activated plasma, and plasma/platelets) induced any marked elevation of the IL-8 levels. IL-8 production on the bioinert pristine and plasma/platelets-coated Ti-PS and ZrO_2_ surfaces was high (60, 90%) in comparison with the osteoconductive surfaces as well as with TCPS. On the other hand, the serum coating decreased the IL-8 production to 19% and the activated plasma coating induced a low IL-8 expression below 10% comparable with TCPS.

EGF production (Figure 5(a)) was similarly high (70–90% of the positive array control) and showed trends as observed for IL6, GRO-*α*, and GRO cytokines. Only the rough pristine and serum coated Ti-PS and serum coated Ti-HA surfaces induced a half as large EGF expression than the other surfaces. The most uniform production was observed for the plasma/platelets coatings.

Minor traces of GM-CSF (Figure 5(b)) were detected on TCPS and all the Ti-Etch surfaces. On the other hand, production on Ti-PS surfaces reached approximately 80% of the positive array control. In the case of ZrO_2_ ceramics, the activated plasma and plasma/platelets coatings almost completely suppressed GM-CSF production in contrast to high values (90%) observed for pristine and serum coated ZrO_2_. Furthermore, a high production (90%) observed on pristine Ti-HA was reduced to traces by the serum or activated plasma coating.

The MCP-1 (CCL2) chemokine was expressed in low doses below 10% on the bioinert pristine and activated plasma-coated Ti-PS and ZrO_2_ surfaces and moderately increased when these surfaces were coated with serum and plasma/platelets (20 and 40%). The MCP-1 production was not consistent on the osteoconductive materials: Ti-Etch showed a low production below 10% on all the surfaces but the serum coated one (80%) and high production (85%) was observed only on the pristine and activated plasma-coated Ti-HA (Figure 5(c)).

## 4. Discussion

Implant insertion is a (surgical) wound involving artificial material, which triggers a foreign body reaction and represents an immunological and inflammation-driven process with the ultimate end to shield off the foreign material placed in the body [15, 36]. Blood is a site of the first contact between the foreign implant material and the living tissue of a recipient [15, 16]. In this study, we intended to evaluate the effect of particular blood components on proliferation and synthetic activity of osteoblasts and cytokine production of human peripheral blood mononuclear cells cultivated on commonly used implant materials, that is, Ti-HA, Ti-Etch, Ti-PS, and ZrO_2_ ceramic in one complex study. To this end, we extended the basic cellular* in vitro* model of implant integration by deposition of a natural biological layer of blood proteins (albumin, fibrin network) and/or platelets on the implant surface in order to mimic the situation immediately upon implantation. This model was employed to create* in vitro* conditions approaching* in vivo* situation and thus better answer the question how blood components influence or support the initiate phase of the implant healing into the bone.

Since wound healing occurs in the range of hours up to days after injury [15, 49–51], we focused on the early proliferation and immune response of the connective tissue cells (NHOst) and immunocompetent cells (PBMCs) after 6-day cultivation. This period corresponds to the development of foreign body reaction and production of cytokines immediately after implant insertion [15, 50, 52]. The short cultivation time reflects the platelet lifespan of 5–9 days as well [12, 17].

All the pristine materials used in our study are commercially available implant materials and support the growth of osteoblasts (Figure 2(a)). A sufficiently high NHOst proliferation resulted from a combination of several factors such as appropriate polar component *γ* ^*P*^ of Ti-based surfaces, high surface roughness preferred by osteoblasts (Ti-PS, Ti-HA, and Ti-Etch), moderate surface hydrophilicity (Ti-Etch, ZrO_2_ ceramic), or biotolerance [3]. Proliferation increased in the Ti-PS → ZrO_2_→ Ti-HA → Ti-Etch line. The trend in cytokine expression was inversed to the proliferation trend; cytokine production increased in the Ti-Etch → Ti-HA → Ti-PS → ZrO_2_ line (Figures 2(b), 2(c), and 2(d)) and was markedly higher than that in the TCPS control.

The serum coating did not affect NHOst proliferation on Ti-Etch, Ti-PS, and ZrO_2_ in comparison with the pristine surfaces. The serum coating also did not alter BAP or IL-8 expression. Proliferation significantly increased only on Ti-HA and this resulted in a lower BAP and IL-8 production. On the other hand, RANKL expression was at least twice as half as that observed on all the pristine surfaces. Elevated RANKL production leads to the initiation of bone resorption which is required for successful bone remodeling* in vivo* [53].

The plasma/platelets coating, similarly to the serum coating, had no effect on the osteoblast proliferation on the Ti-Etch, Ti-PS, and ZrO_2_ surfaces. The explanation for the unaffected proliferation could be insufficient platelet concentration used or their low activation [54, 55]. The ZrO_2_ ceramics showed the lowest number of platelets (Figure 3(a)) and serotonin production was even lower than in the TCPS control (Figure 3(c)). The finding for ZrO_2_ correlates with our previous results, where almost no platelets adhered to the ZrO_2_ ceramics with very low surface roughness [3]. The number of platelets that adhered to the pristine surfaces increased in the ZrO_2_→ Ti-HA → Ti-Etch → Ti-PS line (Figure 3(a)) but the platelet activation on Ti-HA, Ti-Etch, and Ti-PS measured by serotonin production was comparable (app. 125% of the TCPS control, Figure 3(c)). The plasma/platelets coating had a significantly positive effect on NHOst proliferation only on Ti-HA (Figure 2(a)). The observed high osteoblast proliferation could result probably from the activation of platelets by Ca^2+^ ions from the HA layer [56]. Concerning the synthetic activity, likewise as we observed for the serum coating, the plasma/platelets coating had no supporting effect on NHOst cell differentiation and IL-8 production, whereas, contrary to the serum coating, RANKL production slightly decreased. The exception is the bioinert ZrO_2_ ceramic; this surface induced low platelets activation; therefore, one can assume low production of OPG by platelets which is known to decrease RANKL expression [57]. A low effect of the plasma/platelets coating on osteoblast behavior observed in our study can be correlated with the results of clinical and experimental studies using PRP or even additionally activated PRP in form of the gel (PRG) for augmentation of bone healing process. The positive effect of PRP or PRG on healing process is very variable and often unconfirmed [7, 14, 18–22] probably as a result of different activation protocols, anticoagulant effects, or study designs [6, 11, 12].

Contrary to serum and plasma/platelets coatings, the coating with the activated plasma, that is, fibrin network (Figure 1), led to surfaces with a high proliferation rate comparable with TCPS regardless of the surface origin. The favorable conditions for cell proliferation resulted in suppressed differentiation cell activity (Figure 2(b)). RANKL production was markedly enhanced as we also observed for the serum coating. This coating also inhibited IL-8 production especially on the bioinert Ti-PS and ZrO_2_ what was not observed for the serum and plasma/platelets coatings. The overall unification of the surfaces coated by the fibrin network could be advantageous for the coating of bioinert or biotolerated implants (e.g., titanium alloys, C/C composites) which are favorable from a biomechanical point of view.

The spectrum of the expressed cytokines depends on the secretion ability of the tested cells [15, 49], physicomechanical surface properties [15, 52], coatings, or cultivation time [12, 52, 58]. An appropriate evaluation of cytokine production can be obtained by cytokine profiling using simultaneous detection of cytokines [59]. In our study, we used the RayBio Human Cytokine Antibody Array 3 method to screen 40 different cytokines (Figure S2) in cultivation media of PBMCs cultivated on the tested surfaces.

The detected cytokines IL-6, GRO-*α*, GRO, ENA-78, IL-8, GM-CSF, EGF, and MCP-1 belong to cytokines of the early inflammatory phase of wound healing [15, 49–52, 58]. Their presence indicates that an innate immunity reaction has been triggered via the cell contact with the implant surface and the wound healing process started [15, 49–51]. Since we did not prove typical inflammatory cytokines such as IL-1*β* or tumor necrosis factor-*α* (TNF-*α*), we can assume that the intensity of the immune reaction is lower than that observed for ordinary injuries.

Cytokine profiles observed for all the pristine and coated materials were almost identical. These profiles correspond to the cytokine profile detected for different types of injuries with no respect to the injury origin (e.g., burn, infection, and wound) [58, 60]. Our observation correlates with the bioinert/osteoconductive character of the pristine surfaces and with the fact that blood is a natural and essential part of wound healing. We found differences in the cytokine expression mainly for growth factors (EGF and GM-CSF), CXC family chemokines (IL-8 and ENA-78), and CC family chemokine (MCP-1) produced within the inflammatory phase of wound healing; all these differences reflect individual properties of the pristine materials and biological coatings.

Comparable production of the main inflammatory cytokines of the early inflammatory phase of wound healing (IL-6 cytokine, GRO, and GRO-*α* chemokines) for all the surfaces indicates (Figures 4(a), 4(b), and 4(c)) that the extent of immune response to the implantation material is caused by the presence of a foreign body rather than distinctive differences in the surface character or its modification. A high IL-6 expression on the activated plasma and plasma/platelets-coated TCPS surface resulted from stimulation of IL-6 expression by fibrin or its degradation products [61]. Indeed, a negligible IL-6 production was observed on the pristine and serum coated TCPS where no fibrin was present.

Similar differences were found for the epidermal growth factor (EGF) expression (Figure 5(a)). Its upregulation after an acute injury significantly accelerated reepithelialization and increased tensile strength in wounds [49]. On the other hand, a decreased EGF expression was detected in chronic wounds [51]. Similar higher values were detected for all the tested materials coated with plasma/platelets and activated plasma (except for Ti-Etch). Our findings correspond to the* in vivo* experiments where PRP application often supported the healing process [12, 18, 62] probably due to a growth factor release from the platelets [49]. On the contrary, the surfaces inducing lower EGF production, that is, the pristine and serum coated Ti-PS and the serum coated Ti-HA, represent bioinert surfaces or coatings with an inactive biological layer.

Differences in IL-8 production correspond with the biological properties of the implant materials. The reason could be a wider effect of IL-8, which not only consists in attraction of neutrophils into the site of injury but also regulates the other wound healing phases such as reduction of the number of fibroblasts during wound contraction, which begins in the proliferative phase and continues during remodeling [63]. The profibrotic effect of IL-8 was also described. In our study, IL-8 production on osteoconductive pristine Ti-HA and Ti-Etch was low (Figure 4(e)) with no respect to the coatings. On the contrary, PBMCs produced a significantly higher amount of IL-8 on bioinert pristine and plasma/platelets-coated Ti-PS and ZrO_2_. This observation corresponds with clinical data. Bioinert implants without a surface treatment reach a lower resistance to removal of torque and therefore require a longer healing period before loading than osteoconductive ones [64] and cannot be used for an immediate implant loading concept [65]. Healing is often enhanced by such bioactive procedures as the use of the PRP coating [12, 18, 62, 66]. High levels of IL-8 chemokine accumulate in nonhealing wounds [49]. In our study, IL-8 production by PBMCs cultivated on the pristine surfaces increased in the Ti-Etch → Ti-HA → Ti-PS → ZrO_2_ line. The identical trend was observed also for NHOst (Figure 2(c)) and was inversed to the proliferation rate of osteoblasts. This observation can be explained by the fact that the inflammation phase did not provide convenient conditions for the cell proliferation.

Our study implies that when analyzing the results of* in vitro* evaluation of implant materials it would be convenient to take into account the notion that significant changes in the characteristics of cellular behavior at the implant/tissues interface can occur when applied* in vivo*. For example, the fibrin coating used in our study to simulate the blood clot formation in the surgical wound between the bone and the implant can lead to the surfaces with the comparable osteoblast proliferation or BAP and IL-8 production regardless of the cell proliferation rate and synthetic activity on the pristine surfaces. Immunocompetent cells produced cytokines of the initial inflammatory phase of wound healing (IL-6, GRO-*α*, or GRO) irrespective of the type of the blood component coating. On the other hand, the expression of other cytokines, such as ENA-78 or IL-8 (cytokines of later inflammatory phase), was material and/or coating dependent. Regarding the clinical practice, our results imply that the application of platelet concentrate preparations would be more effective for bioinert implants than for bioactive ones with respect to enhanced osteoblast proliferation and decreased production of proinflammatory chemokines. Bioinert implants are used in clinic in case of patients with hypersensitivity to metals (ZrO_2_) or for load bearing applications (e.g., plasma-sprayed titanium alloys implants).

## 5. Conclusion

Implant insertion is a wound with the presence of an artificial material and therefore the blood is a site of the first contact between the foreign implant material and the living tissue of a recipient. In this study, the implant materials Ti-plasma-sprayed (Ti-PS), Ti-etched (Ti-Etch), Ti-hydroxyapatite (Ti-HA), and zirconium oxide ceramic (ZrO_2_) were coated by three blood components, specifically serum, plasma/platelets, and activated plasma (resulting in formation of a thin fibrin network), to imitate better the real environment in the wound in* in vitro* conditions. We studied how the particular blood components affect proliferation and synthetic production of normal human osteoblasts and cytokine production of peripheral blood mononuclear cells in comparison with the pristine surfaces.

The serum coating did not support proliferation and differentiation of the osteoblast cells as well as production of proinflammatory mediator IL-8. On the other hand, RANKL production markedly increased. The coating with plasma/platelets supported osteoblasts proliferation only on the osteoconductive Ti-HA and Ti-Etch surfaces. However, differentiation and IL-8 and RANKL production remained unaffected or even slightly decreased. The coating with the activated plasma led to the formation of uniform surfaces regardless of the surface origin. The proliferation was significantly increased and comparable with the TCPS control in contrast to decreased cell differentiation and IL-8 production. Cell osteoclastic activity (RANKL) was markedly enhanced.

Among 40 tested cytokines, peripheral blood mononuclear cells produced only cytokines involved in inflammatory phase of wound healing, that is, IL-6, GRO-*α*, GRO, ENA-78, IL-8, GM-CSF, EGF, and MCP-1. Compared with the noncoated (pristine) surfaces, none of the biological coatings significantly affected a high production of IL-6, GRO, and GRO-*α*. The serum coating did not alter a generally high production of EGF observed on all the surfaces, except the marked decrease on Ti-HA of 50%. IL-8 production decreased on the bioinert Ti-PS and ZrO_2_ whereas ENA-78 expression considerably increased only on the hydrophobic Ti-HA and Ti-PS surfaces. When surfaces were coated with plasma/platelets, EGF and IL-8 expression remained unchanged, but ENA-78 production increased on the bioinert Ti-PS and ZrO_2_. The coating with the activated plasma provided reduced EGF expression on Ti-Etch and increased one on Ti-PS; ENA-78 production increased on the hydrophobic Ti-HA and Ti-PS similarly to the serum coating. This coating resulted in a very low uniform production of IL-8. High Il-8 levels decreased to one-tenth of the pristine surfaces especially on the bioinert Ti-PS and ZrO_2_. To summarize, compared with the noncoated pristine surfaces, the biological coatings did not alter production of IL-6, GRO, GRO-*α*, and EGF and significantly decreased IL-8 expression (especially the activated plasma) on all the surfaces and promoted ENA-78 production on the hydrophobic surfaces.

To conclude, the activated plasma coating provided uniform surfaces with high osteoblast proliferation and low production of the proinflammatory IL-8 chemokine by both osteoblast and mononuclear cells. These findings could be advantageous for the coating of biotolerant implant materials favorable from a biomechanical point of view. Moreover, in the case of patients with hypersensitivity to dental metallic materials, the only option is ZrO_2_ ceramic. The coating by the activated plasma could improve ZrO_2_ biological properties to the level of bioactive materials in a clinical practice.

## Supplementary Material

Clotting time of human citrate-phosphate-dextrose stabilized blood plasma. The clotting time of human citrate-phosphate-dextrose stabilized blood plasma (CPD, IHBT, Prague, Czech Republic) was estimated by a turbidity measurement on the TCPS control surface as follows: calcium chloride (10 mM) was added to the freshly thawed CDP plasma. 1mL of the solution was immediately transferred to the polystyrene cuvette (1 cm path length) for measurements. The intensity changes of the light passing through the cuvette were measured at 350 nm in two-minute intervals at the room temperature using a spectrophotometer (Biochrom 3 Libra S22, Cambridge, UK). The results of the turbidity measurement are presented in Figure S1. Figure S1: Coagulation of citrate-phosphate-dextrose stabilized blood plasma initiated by Ca^2+^ cations. (turbidity measurement, λ = 350 nm). (Supplementary information) Figure S2: RayBio® Human Cytokine Antibody Array 3 Map.

## Figures and Tables

**Table 1:** Selected surface characteristics of implant materials: the surface free energy (*γ*) and its polar (*γ* ^*P*^) and disperse (*γ* ^*D*^) parts, advancing (*θ*_*A*_) and receding (*θ*_*R*_) contact angles, and the surface roughness (*R*_*a*_).

Surface	*γ* ^*P*^ (mN/m)	*γ* ^*D*^ (mN/m)	*γ* (mN/m)	*θ*_*A*_ (degrees)	*θ*_*R*_ (degrees)	*R*_*a*_ (*μ*m)
TCPS	27.06 ± 1.11	43.15 ± 2.53	70.21 ± 1.92	65 ± 3	43 ± 2	NA
Ti-PS	NA^*∗*^	NA^*∗*^	NA^*∗*^	110 ± 3	3 ± 1	14.51 ± 2.74
Ti-Etch	9.44 ± 2.74	35.56 ± 2.13	43.01 ± 4.62	64 ± 6	33 ± 4	3.54 ± 0.06
ZrO_2_	6.78 ± 1.08	34.56 ± 1.53	41.34 ± 1.91	70 ± 4	15 ± 3	0.85 ± 0.02
Ti-HA	4.62 ± 0.07	2.61 ± 0.01	7.23 ± 3.05	96 ± 8	10 ± 3	7.73 ± 0.49

^*∗*^NA: not analyzed; for comment, see Section 3.1.
