# Supplementary material for: Effect of Blood Component Coatings of Enosseal Implants on Proliferation and Synthetic Activity of Human Osteoblasts and Cytokine Production of Peripheral Blood Mononuclear Cells
Source: Mediators Inflamm. 2016 Aug 29;2016:8769347. doi: 10.1155/2016/8769347 (PMC5019932; doi:10.1155/2016/8769347)
Supplement: Supplementary file 1 — Clotting time of human citrate-phosphate-dextrose stabilized blood plasma. The clotting time of human citrate-phosphate-dextrose stabilized blood plasma (CPD, IHBT, Prague, Czech Republic) was estimated by a turbidity measurement on the TCPS control surface as follows: calcium chloride (10 mM) was added to the freshly thawed CDP plasma. 1mL of the solution was immediately transferred to the polystyrene cuvette (1 cm path length) for measurements. The intensity changes of the light passing through the cuvette were measured at 350 nm in two-minute intervals at the room temperature using a spectrophotometer (Biochrom 3 Libra S22, Cambridge, UK). The results of the turbidity measurement are presented in Figure S1. Figure S1: Coagulation of citrate-phosphate-dextrose stabilized blood plasma initiated by Ca2+ cations. (turbidity measurement, λ = 350 nm). (Supplementary information) Figure S2: RayBio® Human Cytokine Antibody Array 3 Map. [file 8769347.f1.pdf]

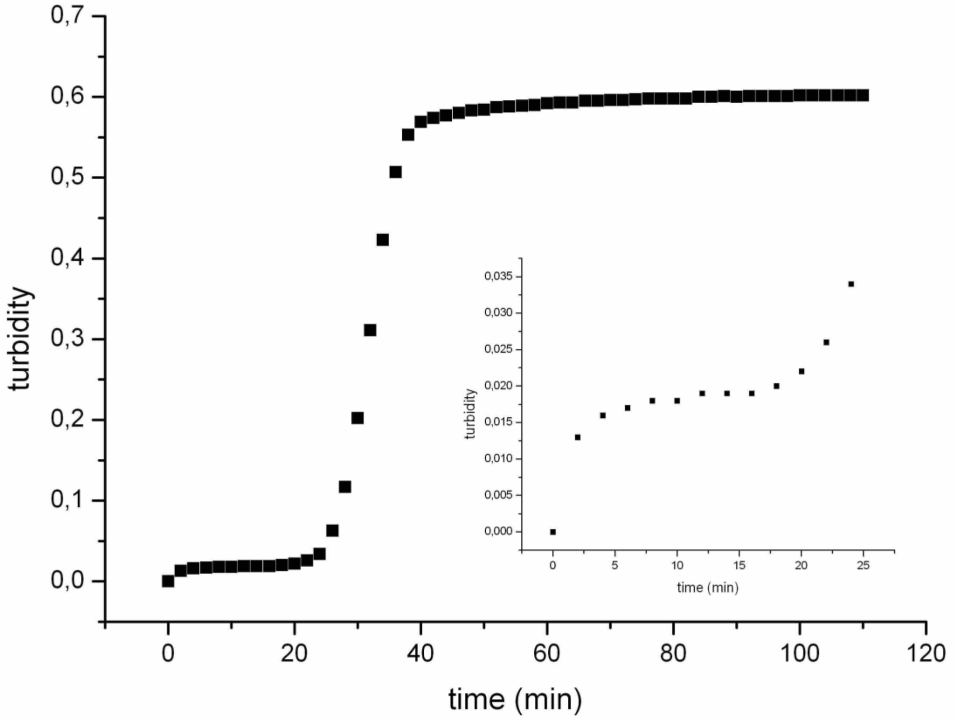

turbidity
time (min)
0,7
0,6
0,5
0,4
0,3
0,2
0,1
0,0
0
20
40
60
80
100
120
turbidity
time (min)
0,035
0,030
0,025
0,020
0,015
0,010
0,005
0,000
0
5
10
15
20
25

	a	b	c	d	e	f	g	g	h	I	j	k
1	Pos	Pos	Neg	Neg	ENA-78	GCSF	GM-CSF	GRO	GRO-α	I-309	IL-1 α	IL-1 β
2	Pos	Pos	Neg	Neg	ENA-78	GCSF	GM-CSF	GRO	GRO- α	I-309	IL-1 α	IL-1 β
3	IL-2	IL-3	IL-4	IL-5	IL-6	IL-7	IL-12 p40p70	IL-10	IL-8	IL-13	IL-15	IFN- γ
4	IL-2	IL-3	IL-4	IL-5	IL-6	IL-7	IL-12 p40p70	IL-10	IL-8	IL-13	IL-15	IFN-γ
5	MCP-1	MCP-2	MCP-3	MC-SF	MDC	MIG	MIP-10	RANTES	SCF	SDF-1	TARC	TGF- β 1
6	MCP-1	MCP-2	MCP-3	MC-SF	MDC	MIG	MIP-10	RANTES	SCF	SDF-1	TARC	TGF- β 1
7	TNF- α	TNF- β	EGF	IGF-I	Angiogenin	Oncostatin M	Thrombopoietin	VEGF	PDGF BB	Leptin	Neg	Pos
8	TNF- α	TNF- β	EGF	IGF-I	Angiogenin	Oncostatin M	Thrombopoietin	VEGF	PDGF BB	Leptin	Neg	Pos
